# Impact of Subliminally Presented Words Valence’ on Risk-Taking Decisions in a Game of Chance

**DOI:** 10.3389/fpsyg.2019.00959

**Published:** 2019-04-30

**Authors:** Maciej W. Pastwa, Kamil K. Imbir

**Affiliations:** Faculty of Psychology, University of Warsaw, Warsaw, Poland

**Keywords:** affect, valence, subliminal verbal stimuli, risk, decision-making, game of chance

## Abstract

A positive mood is thought to accompany performing a risk-taking tendency, for instance in games of chance or gambling. This study concerns the impact of emotional stimuli presented in a subliminal manner on the riskiness of decisions made in a game of chance. The heights of stakes called in the game were adopted as the measure of risk taken. Special simulation of a real game of chance, based on coin tossing, was used for this experiment. The stimuli displayed subliminally were words differing in valence (three levels: negative, neutral, and positive). We expected that positive valence would provoke the riskiness of the subsequent decision. The main effect of the valence observed was that the subjects in positive word conditions bet higher stakes than in neutral and negative conditions. Positive emotions influenced the riskiness of decisions made by the subjects, which confirmed the set hypothesis. The results of the study, in addition to their theoretical implications, may have practical meaning due to realistic simulation of popular games of chance.

## Introduction

In today’s world there is a lot of information that reaches each of us from different sources, therefore it is difficult to verify what has the greatest impact on decision-makers. Furthermore, the information that affects decision-makers often has nothing to do with the actual outcome of the decisions made. However, there are situations where it is virtually impossible to predict the result of the decision – when it is purely random. Chance and gambling games are perfect examples of such situations. Despite random conditions, the same people, in different situations, tend to play such games in either a risky or a safe way. In this article we tried to explore the relation between emotions and decisions made in this kind of game ruled by randomness. In particular, we were interested in incidental sources of affect, including emotional reactions to stimuli not consciously recognized.

### Valence of Emotion and Verbal Affective Priming

Valence is a specific factor that characterizes emotions, which can be intuitionally understood by people (Kagan, 2007; Kousta et al., 2009). Frijda (1986, 1993) divided emotions into three basic components, outlining the evaluation of the stimulus as one of them (other two were subjectively experienced state and the triggered behavioral response. The evaluation leads to labeling the stimulus on the valence scale as positive or negative. The concept of emotional valence has been further developed in later studies, standing now as the most basic dimension that can describe emotions (Prinz, 2004; Oatley et al., 2006).

Emotions, characterized by their valence, are in fact a kind of evolutionary signpost that signals sympathy or antipathy for a given stimulus. Studies show that when recognizing facial expressions presented on pictures, the pleasant–unpleasant dimension is responsible for more than 50% of the variance of results (Abelson and Sermat, 1962; Hastorf et al., 1966; Ekman et al., 2013). The emotional component appears at a very early stage of stimulus processing, which has been proven frequently. For example, we can recognize the intention of the sender of verbal communication without understanding the actual message (Dawes and Kramer, 1966; Scherer et al., 1972; Sauter et al., 2010); the tone of voice is much more important for understanding the content of communication than the content itself (Argyle et al., 1970; Jacob et al., 2014). Relying on the above-described relations, as well as examples from life, such as the fact that the emotional component is an inseparable element of consumer decisions, Zajonc (1980) introduced the theory that giving the stimulus emotional valence appears before its semantic recognition. Processing on the emotional level is, according to him, a parallel process that occurs at all levels of semantic recognition.

Describing these characteristics of emotions led to using emotionally charged stimuli in experiments with an ultra-short time of exposure. This paradigm, called subliminal affective priming, was introduced by Murphy and Zajonc (1993) and its effectiveness has been confirmed repeatedly (Banse, 1999; Koole and Coenen, 2007; Hinojosa et al., 2009). A typical experiment conducted in the paradigm of subliminal affective priming consists of preceding the neutral stimulus with an emotional stimulus. In the original study Chinese ideograms were the neutral stimuli that, for English-speaking people who did not speak Chinese, did not arouse positive or negative associations (Murphy and Zajonc, 1993). Faces expressing positive emotions were used as the affective stimuli. Chinese ideograms were appraised as more positive when they were preceded by smiling faces than when they were preceded by neutral stimuli (black squares). In fact, the valence of emotional stimuli is virtually the most basic characteristic of emotion and is responsible for its induction in subliminal conditions.

The results of the study by Banse (1999) indicate that words are a specific kind of emotionally charged stimulus that, when presented for an ultra-short time, can arouse high affection. In this study, Chinese ideograms, neutral to the group of respondents, were preceded by the name of the partner with whom the examined person remained in a relationship. In the condition of priming for less than 20 ms with the name of the partner, the Chinese ideograms were assessed significantly more positively than in the condition of consciously seeing the partner’s name. In the study by Abrams et al. (2002), subjects classified word stimuli into two categories – pleasant and unpleasant – under optimal presentation conditions. Later, the words classified by the respondents were displayed subliminally before the presentation of neutral stimuli in optimal conditions. The subjects tended to classify these neutral stimuli according to the category assigned to the preceding word stimulus. Analogous results were obtained in another experiment (Ferguson et al., 2005) where the words were classified as positive or negative. The results show that the subjects tended to rate the word as positive if it was preceded by a positive stimulus displayed for an ultra-short time. A recent meta-analysis (Weingarten et al., 2016) described 133 studies that include words as experimental stimuli, providing very strong presumptions for using words in this paradigm. Taking this into account, we may argue, that affective charge may be transmitted by the affective meaning of words (Banse, 1999; Zhang et al., 2012). Brief presentation of words leads to elicitation of an implicit affective response that may be treated as a model of incidental affect appearing in real-life situations (Russell, 2003).

### Risk-Taking and Emotions

It seems intuitively clear that primal affective states should have an influence on every aspect of human life, including decisions regarding level of risk taken. Although this interaction seems obvious, its details still remain not sufficiently described (Lupton, 2013). If we focus on the danger involved in the risk, then risk-taking will be any action that can bring losses, and the magnitude of the risk will be understood as the actual or imagined amount of loss (Shapira, 1994). Regardless, actual risk may not be considered without the level of risk perceived by the object (Slovic, 1967).

Kahneman and Tversky (2013) and Kahneman (2003) proposed Prospect Theory to explain the role of perspective in the decision-making process. Emotional state may give some kind of framework for the decision-making situation. For instance, people present a different risk propensity depending on whether they assume that they operate under conditions of loss (loss seems more likely) or profit (profit seems more likely). The less reliable but more profitable option is chosen more often in the case of profit than in a loss situation (Kahneman and Tversky, 2013). Neurobiological evidence suggests that the impact of losses may be stronger than the impact of profits when making a decision – losses arouse a stronger response from the autonomic nervous system (ANS; Hochman and Yechiam, 2011). Furthermore, the response from the ANS is correlated with the perception of risk and not with the propensity to take risk itself.

When it comes to emotions, we may assume that positive affect is related to the situation of profit, whereas loss would be connected with negative emotions. Studies regarding relations between emotions and cognitive processes suggest that emotional stimuli prolong the latencies for processing cognitive stimuli (Gupta and Raymond, 2012; Gupta et al., 2018). There is also a widely described relation between the valence of emotion and the scope of attention. Positive emotions broaden the scope of attention, whereas negative emotions promote focused, local attention (Srinivasan and Gupta, 2010, 2011; Gupta and Deák, 2015; Gupta and Srinivasan, 2015; Gupta et al., 2016). The global scope of attention is related to exploring: the search for stimulation and similar inquisitive behavior. Thus, we can conclude that positive emotions that promote play-alike behavior may lead to more risky decisions (Fredrickson, 2004). This relation may take place especially in the situation of a game of chance, as more risky behavior gives the opportunity for a longer and more stimulating game.

The obvious motivation leading to engaging in chance and gambling games seems to be the possibility of winning money, but gamblers declare that they get involved in the game mainly for pleasure (Anderson and Brown, 1984). One may doubt the sincerity of such declarations, however, the results of research using neuroimaging dispel these doubts. Commitment to gambling stimulates areas in the brain responsible for pleasure (Potenza, 2001). This pleasure is not only related to the expectation of winning but is also directly related to the risk-taking situation. Bateman et al. (2007) compared the attractiveness of two games of chance: the first one allowed subjects to win a certain amount or simply not win anything; in the second one there was the possibility of sustaining a small loss. The second game turned out to be more engaging for the respondents. The study by Mellers (2000) also indicated that expected pleasure may be one of the main factors responsible for making risk-taking decisions. In Antonio Damasio’s research (Damasio, 2005) two groups of patients with cerebral lesions participated in an experiment: subjects from the first group were deprived of that part of the brain responsible for feeling emotional arousal (lesions in the *amygdala*), whereas patients from the control group had these structures intact. During the experiment subjects participated in a gambling game. Patients not able to feel emotions played more riskily and bet higher stakes but also achieved higher profits than patients from the control group. Emotions should undoubtedly be considered an important factor in evaluating and taking risks.

In an interesting study, business school students were asked to freely evaluate various options for investing money on several scales. Some of the scales concerned sympathy for the industry and there were also questions about willingness to invest in the given option or its expected profitability. The possibilities that received higher rates on the affective scales were also rated as more profitable, and the respondents also were more willing to invest in them. Thus, emotional attitude to the choice has an impact on decision-making, even among members of the expert group, who, in the assumptions, should rely only on the cognitive evaluation of possibilities (MacGregor et al., 2000). An analogical relationship was found in a similar study conducted on a group of experienced financial analysts. They rated, on certain scales, how specific investment options in a decision-making situation increase their level of anxiety. The possibilities that received high scores on the scale of anxiety were less frequently chosen for investment (MacGregor et al., 1999).

In the study by O’Neill et al. (2008), business school students first received a specially prepared letter to shareholders that, depending on the experimental condition, presented positive or negative information about the company’s achievements. Then, playing the role of the company’s manager, they had to decide on the amount of investment in the sector of research and development. It is worth noting that the invoices in this area are considered to be the most risky, due to the long period over which they can bring profits. The negative information presented in the letter to shareholders significantly affected the lower value of the declared investment in the sector of research and development. Similar results were found in another study (Erb et al., 2002), where subjects simply assessed the attractiveness of different consumer choices after their moods were manipulated. According to the results presented previously, options considered as more exciting were chosen after an induction of positive affect, whereas in the negative state rather calm options were preferred. The same relations may be described when it comes to declarative measures without an engaging experimental situation. Subjects under the influence of positive affect tended to choose more risky options than when under the influence of negative affect (Hu et al., 2015) when filling out a questionnaire containing hypothetical risk-related decisions. It is therefore particularly interesting to examine the impact of emotions on the propensity to risk under conditions of controlled variance of positive and negative results known to the decision-maker at all times. This kind of task would allow outlining the psychological factors responsible for propensity to risk. Such conditions are specific to games of chance such as roulette or lotto, where the result is random, the weight of each option is the same, and decisions made by the players can still differ in the risk taken, operationalized as the height of the stake.

In an interesting study, a differently valenced affect was induced in a natural-like way – the subjects got to know the results of their exams (positive or negative) and then played risk-related games (Balloon Analog Risk Task and Iowa Gambling Task). Subjects under the influence of negative emotions (negative exam result) made less risky choices in these games than under the influence of a positive affect (Heilman et al., 2010). In the study by Wohl et al. (2014), surveyed students in the experimental group read an article about a difficult situation in the labor market and bad prospects for students finishing universities. It can be assumed that such a text evoked negative emotions in the subjects: most likely fear. Then the subjects had the opportunity to play a gambling game – roulette. The results of the experiment show that the subjects from the experimental group bet higher stakes than those in the control group. These results agree with those from another study where participants were induced with the emotion of fear (Kugler et al., 2012). Subjects were instructed to describe vividly a specific situation in which they experienced fear or anger. Subsequently, they were introduced to a specially prepared lottery task in which they had to choose between options characterized with different levels of risk. The fearful ones preferred to choose risk-avoiding options more frequently than the anger-primed ones.

Priming the target risk-related decision can be done under supraliminal conditions (when the priming stimulus is seen and well registered by the subject) and subliminal conditions (when the priming stimulus is not consciously registered by the participant). Subliminal priming is more likely to evoke an assimilation affect between the prime and the target (Bargh and Pietromonaco, 1982; Wróbel and Imbir, 2019). In an interesting study (Gibson and Zielaskowski, 2013), a subliminal stimulus was used particularly to prime risk-taking decisions. Subjects had an opportunity to play a specially prepared gambling game. The game apparently did not differ from popular games such as “one-armed bandit” except for one small detail: the results of the game appeared on a screen. From time to time the screen showed a “777” image (meaning the highest win) for 30 ms. It is worth adding that the subjects were previously instructed about the meaning of symbols used in the game, thus the symbols shown could have a strong positive charge as one indicating very high profit. People from the experimental group, to whom the subliminal stimuli were presented, were betting significantly higher stakes than those from the control group. The second experiment from this study not only confirmed the result but also found that the relation only occurs if the person chooses the height of the stake immediately after presentation of the subliminal stimulus. In this experiment an in-game-specific stimulus was used. We were particularly interested in investigating the influence of generally emotional stimuli under similar conditions. Differently valenced words, mentioned in the previous parts of this article, seemed to be a perfect fit, as the impact of a three-digit number (which in fact is a series of symbols) has been proven already.

### Aim and Hypothesis

In real life the affect accompanying gambling is evoked most often by the contextual stimuli (smells, music, surrounding colors, etc.). This affect is an impulse lasting milliseconds or seconds rather than a long-term state. If such an impulse arises at a critical moment in the decision-making process, it may have a significant impact on the decision. In the current experiment, we wanted to be as close as possible to the natural situation We wanted to make the simulation of the game of chance as realistic as possible, having the participants convinced that they are playing an actual game. Therefore, we decided to evoke the affect subliminally, without conscious recognition by the participants. Presenting the words supraminally would mean introducing a new part of the procedure that explains the appearance of words, consequently breaking the illusion of a real game of chance. Taking this into account, the affective priming paradigm gives us a unique chance to control the moment of affect elicitation, therefore is useful for studying the impact of affect on risk-taking.

The literature mentioned in the introduction described many experiments where different stimuli were used to evoke affect. Most of the stimuli were specific to the task used or the knowledge of the participants. In our study we decided to prime the decisions with emotionally charged words grouped according to the type of emotions they relate to. Stimuli well known to the whole Polish-speaking population, that has precisely described emotional characteristics (Imbir, 2016), could give us opportunity to conclude about the shape of real-life relation between implicit affect and presented risk propensity.

On the basis of the literature, we predicted that people under the influence of positive affect are more prone to take play-alike and risky decisions than people in a state close to neutral. Earlier studies had shown that when financial risk is concerned in conditions of random outcome, positive emotions predispose people to take risk. We may conclude that a negative state should decrease the risk propensity. In this paper we wanted to answer the question: What would be the influence of emotions of different valence, evoked by subliminal stimuli, on risk-taking?

We also controlled win or loss preceding the experimental trial because it also appeared as a factor that may influence both affective state and the decision made. Regardless of the height of the stake, we were also measuring the decision-making time. We did not state any particular hypothesis regarding these two variables as we did not find enough presumptions in the literature to do so. The main reason for recording data on these variables was to control the process of the experiment and track any hypothetical artifacts influencing the results (e.g., very short reaction times, which could suggest that the game was not engaging for the subjects, or very long reaction times, suggesting some disruption of participants’ attention).

## Materials and Methods

### Participants

A total of 50 participants took part in the experiment (30 males and 20 females), aged from 20 to 29 years old (*M* = 24.4; *SD* = 2.04). All subjects were university students (social sciences, economics, logistics; 10 studied psychology) and they took part in the experiment on a voluntarily base. All of the participants were native Polish-language speakers with normal or corrected-to-normal vision. Data from two subjects were excluded from further analysis (both male) as they claimed they saw the stimulus words during the task and could recall a few of them (consciousness of manipulation test). The final sample consisted of 48 participants aged 20–29 years (*M* = 24.35; *SD* = 2.07).

To verify whether the group of participants was large enough to test the hypotheses, we conducted power analyses for the experiments using G-Power software. We conducted *a priori* analyses using data from different experiments that included a similar procedure (Heilman et al., 2010; Hu et al., 2015). The first of the studies reported a very large effect size (η^2^ = 0.9), which gave the actual power of the study as 0.99 if only five participants were to take part in the experiment. The second of the studies reported a more reliable effect size (η^2^ = 0.22), which gives a power value of 0.95 if 58 participants were to take part. Calculating the mean sample size from these studies suggests that a sample of 32 participants would be large enough to achieve high power for the study. Thus, we concluded that a sample of 48 participants is large enough for statistical analysis.

Participants provided their verbal informed consent to participate in the presence of a lab member and this was documented in a research diary. We did not collect any personal data from our participants to ensure their anonymity. This procedure was suggested by the Bioethical Committee. The design, experimental conditions and consent procedure for this study were approved by the ethical committee at The Maria Grzegorzewska University.

### Materials Used for Experimental Manipulation

Stimuli used to arouse affect in the affective priming part of the procedure were emotional words presented in a degraded way for 32 ms each. The words were taken from the database of Affective Norms for Polish Words Reload (Imbir, 2016) which contains assessments from a normative study on eight different scales: valence (negative vs. positive), origin in the specific emotional system (automatic vs. reflective), arousal (low vs. high), and concreteness (low vs. high) were used to create a factorial manipulation for the current experiment. The frequency of use of these words in the Polish language and the words’ length were also controlled.

The words were divided into three categories of emotion valence (negative vs. neutral vs. positive), with 30 words in each category. The division of the words was done according to certain preset standard assessments from a normative study (Imbir, 2016). Nouns that had ratings on the valence dimension 1 SD higher than the mean rating from all nouns included in the dataset were chosen as the group with positive valence. Accordingly, the words chosen as negative had their ratings on the valence scale at least 1 SD lower than the mean rating for the whole group. The neutral group was created out of words whose ratings were between +0.5 SD and −0.5 SD from the mean rating.

The accurateness of the word selection was checked with six different analyses of variance (ANOVAs). Assessments taken from the normative study for words were compared in groups of words with different levels of valence (three levels: negative vs. neutral vs. positive). As expected, groups labeled with different levels of valence varied on the valence scale [*F*(2,87) = 395.7; *p* < 0.01; η^2^= 0.90] but there were no statistical differences on the other controlled scales: origin [*F*(2,87) = 0.14; *p* = 0.87; η^2^ = 0.003], arousal [*F*(2,87) = 2.42; *p* = 0.095; η^2^ = 0.053], concreteness [*F*(2,87) = 1.19; *p* = 0.31; η^2^ = 0.02], length of words [*F*(2,87) = 0.31; *p* = 0.73; η^2^ = 0.007], or frequency of appearance in the Polish language [*F*(2,87) = 1.51; *p* = 0.23; η^2^ = 0.034]. Means and standard deviations of the above-described ratings in all groups of words may be found in Table 1 and all the exact values of ratings and statistics may be found in Appendix Table A1.

**TABLE 1:** Means and standard deviations for all groups of words on the experimental and control scales.

	Level of valenc							
	Negative		Neutral		Positive		Total	
	*M*	*SD*	*M*	*SD*	*M*	*SD*	*M*	SD
Valence	3.58	0.36	5.16	0.49	6.60	0.39	5.11	1.31
Origin	5.45	1.11	5.60	1.11	5.48	1.32	5.51	1.17
Arousal	4.35	0.48	4.04	0.52	4.16	0.62	4.18	0.55
Concreteness	4.24	1.12	4.02	0.96	4.44	1.12	4.23	1.07
NoL	7.13	2.21	6.87	1.87	7.30	2.31	7.10	2.12
Ln (freq)	5.32	1.64	6.09	1.93	5.87	1.80	5.76	1.80

### Apparatus

The experiment was conducted on a Toshiba notebook with screen dimensions 31.5 cm × 23 cm and monitor rate 60 Hz. The buttons used to play the experimental game were tabbed with colorful stickers.

### Design

The study was planned in a 3 (valence level of words) × 2 (loss or profit situation preceding the decision) factorial design. The dependent variable was the degree of risk taken – operationalized as the height of the stake called in the game of chance. The time to make the decision was additionally measured.

### Procedure

The experimental procedure took place in a laboratory environment and was conducted in individual sessions, all of which took place during the spring. Average time for one session was 30 min. At the start, the experimenter stated that the experiment concerned decision-making in the game of chance. Participants were also informed that each person taking part in the experiment would get a chocolate bar as a small reward. Additionally, participants were told that the person with the highest gain would receive a gift card (shopping voucher): email addresses were shared and used for the voucher lottery, allowing the anonymity of participants to be maintained (results were not matched to a certain person).

Each experimental session started by introducing the field of the study. Subjects were told that the experiment was a simulation of a game of chance and its goal was to examine the perception of the win/loss relation in such a game. They were also told that the whole study was based on a computer program and they should answer using only those buttons with colorful stickers on them. Participants were asked to put their glasses on if they normally use them for the computer.

**FIGURE 1:**
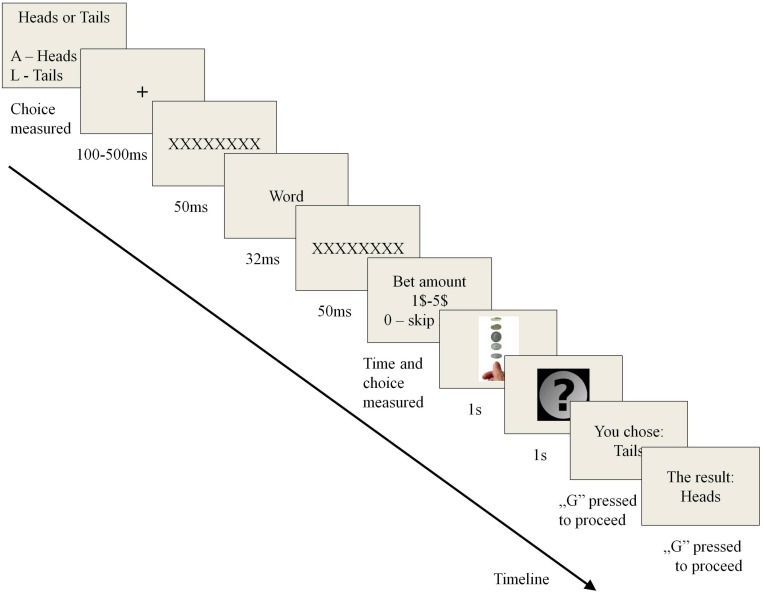
Single trial of “Coin Toss” task (includes priming and decision on bet amount).

As we outlined previously, we wanted to create an experimental procedure that would allow us to measure propensity to take risk immediately after experiencing certain stimuli in fully controllable conditions. In the search for such a procedure the original “Coin Toss” paradigm was created using e-prime software. The procedure was based on a game of chance simulation using the coin toss mechanism. We chose the game of coin toss due to its simplicity and clearness of rules. We also assumed that the choice between sides of a coin is not related to any kind of attitude or magical thinking, which could be possible in games using numbers or cards as objects of choice. First, two screens of the experimental program introduced the game. At the beginning, the participant obtained 100 virtual dollars to be used as the currency for betting in the game (the participant did not profit from the outcome of the game). Next, the subject moved to experimental trials. At the beginning of each trial the participant chose a side of the coin, heads or tails. Next, the screen showed the sequence used for experimental manipulation: fixation cross (random time of presentation between 100 and 500 ms), mask (12 large X letters, shown for 50 ms), stimulus word (one of 90 different words in each trial, shown for 32 ms), mask for stimuli (12 large X letters, shown for 50 ms). The presentation time for subliminal stimuli was based on the time used by Gibson and Zielaskowski (2013), however our apparatus worked at 60 Hz, which makes 32 ms the closest possible option. The words from each of the three categories were shown in random order; each of the six blocks (each valence group was divided into two blocks) was also shown in random order and the order was redrawn for each participant. After manipulation, the subject chose the stake amount called in this trial (0–5 virtual dollars, where 0 meant not betting in this trial). Next, the screen showed two images imitating a coin toss, each for 1 s. After that, the participant was informed about the outcome of the toss (heads or tails), with a note reminding which side was chosen. In each trial the program drew all the outcomes, so they were random in each game. A single trial of the experimental procedure is depicted in Figure 1.

Experimental blocks were separated with questions, which were parts of the mask task. After 15 experimental trials the subjects were asked whether they had been mostly winning or losing (two possible answers: more wins or more losses). The next question considered the participant’s recent award amount (three possible answers: under $100; $100–300; over $300). The aim of this question was also to provide a small break in the procedure, therefore we did not collect or analyze such data. After these questions the subject moved to the next experimental block. After the last mask question a finishing screen with acknowledgments was shown. The log of the program registered the height of the stake called in each trial, the time in which the subject called the stake, and the order of wins and losses.

The average time of one game was 25–30 min. The experimenter was in the same room the whole time in case of any questions from the participant. When the finishing screen of the game appeared, the participant was asked a question: “Have you encountered anything strange while playing the game?”. If the subject answered “yes”, the next question was “what was this strange thing, can you describe it?”. If the subject did not mention words in answer to these questions, he or she was informed that the emotional words were presented subliminally during each trial of the game. The participant was then asked if he or she could recall any of those words. The goal of these questions was to verify whether the experimental word stimuli were seen by the subjects. Next, the experimenter informed the participants about the actual goal of the study and the emotional characteristics of the stimuli used.

## Results

### Preparing Data for Analysis

The data were processed using IBM SPSS Statistics 24 software. A series of operations was performed on the data before the main analysis. All data consisted of records of decisions and reaction times for 48 participants, 90 trials each (4320 trials in total). Data from the first trial for each subject were not analyzed as it was not preceded by any loss or profit (48 trials, about 1% of all trials). One trial was additionally deleted due to an error that occurred in the data. The reaction times from each trial were converted into results that were standardized subject-wise (Z scale). Any trials shorter than 270 ms were deleted as it was assumed that such a period of time was too short for conscious decision in the task (73 trials, 1.7% of all trials). Subsequently, reaction times that differed by about two standard deviations from the mean reaction time were deleted (173 trials, about 4% of all trials). Reaction times were converted into natural logarithms to make the distribution of data as similar as possible to a normal distribution. The above-described treatment of data was conducted to enable statistical analyses specific for normal distribution to be carried out; this would not be accurate for raw latencies (Heathcote et al., 1991; Baayen and Milin, 2010). The final dataset therefore consisted of 4035 trials (trials per subject: *M* = 84.1; *SD* = 3,8; *Min* = 71; *Max* = 88). This means that only 6.6% of all collected trials were excluded in order to achieve greater reliability by eliminating artifacts and erroneous trials. The means of the stakes called in the game were calculated for each subject for two experimental conditions: valence; and loss or profit preceding the decision. The data were analyzed using two-factor analysis of variance in a 3 (valence of emotional charge of the word: negative vs. neutral vs. positive) × 2 (situation of loss or profit preceding the decision: loss vs. win) factorial design.

### Height of Stakes

**FIGURE 2:**
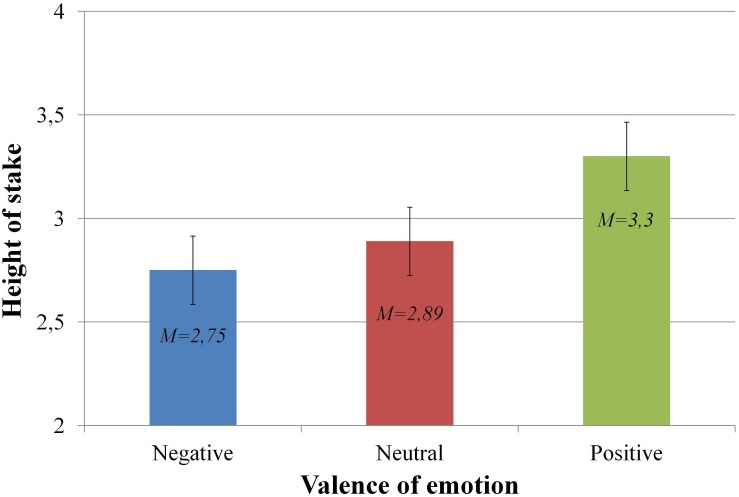
Differences between heights of stakes divided by the emotional charge valence of the preceding stimulus.

Analysis of the height of the stakes called by the participants was conducted. A statistically significant main effect was revealed for the valence of the emotional charge [*F*(2,46) = 9.44; *p* < 0.01; η^2^ = 0.167]. *Post hoc* tests (pairwise comparisons with Bonferroni correction for multiple comparison) showed significant differences in the height of stakes between the positive (*M* = $3.3, *SEM* = 0.14) and negative emotional charge conditions (*M* = $2.75, *SEM* = 0.17) [*t*(1,47)* = −* 3.61; *p* < 0.01; *d =* 0.51] and between the positive and neutral conditions (*M* = $2.89, *SD* = 0.16) [*t*(1,47)* = −* 3.47; *p* < 0.01; *d* = 0.39]. These results are presented graphically in Figure 2. We conducted *post hoc* power analyses for the main effect of valence, based on the effect size achieved in our study (η^2^ = 0.167). The results show a medium power of 0.77 for this experiment.

The preceding profit or loss did not influence the decisions made by the subjects [*F*(1,47) < 0.01; *p* = 0.99; η^2^ < 0.01]. There was also no effect found for interaction between valence and preceding loss or profit [*F*(2,46) = 0.006; *p* = 0.99; η^2^ = 0.001]. All the means and standard deviations of the called stakes for the experimental groups are presented in Table 2.

**TABLE 2:** Means and standard deviations of stakes called according to experimental group (statistically significant differences are marked with * and **).

Preceding loss or profit	Height of stakes ($)					
	Loss		Profit		Total	
Valence	*M*	*SD*	*M*	*SD*	*M*	*SD*
Positive	3.29	1.01	3.30	1.00	3.30${}^{*,}$**	0.97
Neutral	2.91	1.15	2.87	1.17	2.89*	1.12
Negative	2.73	1.26	2.77	1.20	2.75**	1.18
Total	2.98	1.00	2.98	0.97	2.98	0.97

### Reaction Latencies

The natural logarithms of the decision-making times were analyzed. The main effect of valence for this variable turned out to be statistically insignificant [*F*(2,46) = 0.38; *p* = 0.68; η^2^ < 0.01], as well as the effect of the preceding loss or profit [*F*(1,47) = 0.05; *p* = 0.83; η^2^ < 0.01]. There were also no effects found for interaction of valence and preceding loss or profit [*F*(2,46) = 0.41; *p* = 0.67; η^2^ < 0.01]. All the means and standard deviations of reaction times for the experimental groups are presented in Table 3.

**TABLE 3:** Means and standard deviations of reaction times according to experimental group.

Preceding loss or profit	Reaction times (ms)					
	Loss		Profit		Total	
Valence	*M*	*SD*	*M*	*SD*	*M*	*SD*
Positive	1322.73	603.49	1350.53	707.09	1336.63	647.21
Neutral	1320.44	632.83	1353.37	709.99	1336.90	664.90
Negative	1310.13	665.39	1315.00	621.17	1312.56	632.62
Total	1317.76	600.83	1339.64	653.56	1328.70	623.39

## Discussion

The aim of this study was to verify whether and how the valence of emotions affects the propensity to risk when making decisions. The hypothesis about the impact of differently valenced emotions has been confirmed. Subjects under the influence of positive affect made more risky decisions than after neutral stimuli or under the influence of negative affect. We can tell that among the factors controlled in this experiment only valence turned out to be significantly influencing subjects’ behavior.

Results from this experiment are in agreement with those obtained by Erb et al. (2002) and O’Neill et al. (2008). The positive affect evoked by subliminal stimuli encourages making risky decisions in an analogous way to the affect evoked by consciously perceived objects. In the study by Gibson and Zielaskowski (2013), a similar situation with a gambling game was arranged. Assuming that the stimulus presented there (the symbol of the highest win) was interpreted positively, the results of our study confirm the previous findings. In both studies the stimulus was presented subliminally and influenced the decision taken immediately after presentation. Nevertheless, we can only assume that the “777” symbol was interpreted positively because it is actually a cognitive symbol. The materials used for experimental manipulation in our study were words, which contain emotional charge regardless of the specific in-game conditions. It is worth noting that in the presented literature positive stimuli that affected risk-associated decisions were related to the experimental task, while in our experiments emotionally charged words originated from different areas of the language and were not related to the decision or to the whole game.

The relationship between negative emotion and a tendency to risk did not seem obvious. Results from our experiment show that decisions taken under the influence of negative affect did not differ significantly from decisions taken after neutral stimuli, therefore in both these conditions the subjects acted in a more risk-avoiding way than in the positive condition. These results seem to be in accordance with the findings from a study mentioned in this work where fear and anger were induced in the subjects (Kugler et al., 2012), as well as with the relation of negative emotions and risk-taking described by Wohl et al. (2014). Subjects who experienced negative emotions also acted in a slightly more risk-avoiding way in the experiment we carried out.

The observed relation between positive and negative stimuli and risk-taking may be explained by the cognitive mechanism of broadening the scope of attention by positive emotions (Srinivasan and Gupta, 2010, 2011; Gupta and Deák, 2015; Gupta and Srinivasan, 2015; Gupta et al., 2016). In this engaging game situation, exploratory behavior related to positive emotions may be reflected by the more risky decisions. Nevertheless, to fully support this theory, the relation between risky behavior (operationalized as the large amount of invested resources) and inquisitive behavior should be explored.

Decisions taken by the subjects did not differ due to having an earlier win or loss, consequently the outcome of a single trial is probably not able to change the perception of a decision being made in a situation of profit or loss. We also did not find any statistically significant differences between decision times under different conditions (valence; win or loss), which is congruent with our predictions – in such a repeatable and multi-trial game the subjects learned the rules quickly and played at a steady tempo.

The findings of our study fall in with theories about the influence of positive affect on the risk propensity. It is worth noting that the impact of emotions on risk-taking has been observed in strictly controlled and isolated conditions. The amount of information needed to make a decision was minimal, the procedure was very simple, and the variance of profits and losses was random, all of which was acknowledged by the participants at the very beginning of the task. Our results may be particularly accurate due to the elimination of interactive and intermediary variables. The simplicity of the game can also be perceived as a weakness of this experiment, as some of the subjects may not have treated the task as risk-related. Nevertheless, the results show that emotional stimuli not related to the risk-taking situation may affect the decision itself, which is a large difference from other studies in this field of research.

The experiments concerning subliminal stimuli should not be abandoned, despite this field of research seemingly being fully explored. We suggest that relations between words and symbols presented in an ultra-short time and financial or risky decisions should be explored extensively, as today’s digital environments enable these factors to be applied in real-life situations. The field of relations between negatively valenced emotions and decisions concerning risk needs more exploration.

The influence of positive emotions on risk-taking has broad implications in practice. Based on factors influencing the mood of investors worldwide (such as the weather or the political situation), their tendency to take risk during decision-making may be predicted. It should be noted that this is also a trick used in casinos, where keeping a smile and putting customers in a good mood is part of the work of attractive croupiers and hostesses. Casino owners have discovered long ago that a happy player is willing to leave larger amounts of money at the roulette or black jack table. Regardless, the results of our study, as well as other findings presented in this work, bring up a very important issue – subliminal emotional stimuli may be used easily in technologically advanced slot machines and pervasive online gambling. It seems that online gambling and games of chance, as well as slot machines, should be controlled with regard to manipulation of their display, as imperceptible manipulation is indeed able to affect the amounts of money bet in such games.

## Ethics Statement

Participantsprovided their verbal informed consent to participate in the presence of a lab member, which was documented in a research diary. We did not collect any personal data from our participants, to assure their anonymity. This procedure was suggested by the bioethical committee. The design, experimental conditions and consent procedure for this study were approved by the ethical committee at The Maria Grzegorzewska University.

## Author Contributions

Both authors contributed to the final version of the manuscript. KI contributed theoretical proposition and design. MP performed the experimental procedure programming, experiment execution, and figures and tables. MP and KI prepared the statistical analyses, methods, results description, and results discussion.

## Conflict of Interest Statement

The authors declare that the research was conducted in the absence of any commercial or financial relationships that could be construed as a potential conflict of interest.

## Appendix

**TABLE A1:** The list of stimuli used in the experiment with the accompanying affective norms taken from ANPW_R dataset (Imbir, 2016).

Word in	Word in	Group	Group	Valence	Valence	Origin	Arousal	Concreteness	Frequency	Number of
polish	English	(number)	(word)		M	M	M	M		letters
Czkawka	Hiccup	1	Neg	1	4.04	4.54	3.86	3.18	79	7
Szloch	Sob	1	Neg	1	3.04	3.58	4.70	3.68	735	6
Łzy	Tears	1	Neg	1	3.66	3.38	4.54	2.08	660	3
Uszczypniêci	Pinch	1	Neg	1	4.06	4.38	4.38	3.44	24	13
Pijak	Drunk	1	Neg	1	2.76	4.98	5.40	2.84	467	5
Naiwniak	Sucker	1	Neg	1	3.38	4.16	3.84	5.40	23	8
Słabeusz	Weakling	1	Neg	1	3.18	4.98	3.58	5.39	32	8
Zmêczenie	Fatigue	1	Neg	1	3.28	4.90	3.50	5.56	2044	9
Hałas	Noise	1	Neg	1	3.56	4.74	4.60	4.18	3199	5
Plotka	Rumor	1	Neg	1	3.50	4.78	4.48	5.04	588	6
Grymas	Grimace	1	Neg	1	3.76	4.42	4.52	4.20	1618	6
Gafa	Blunder	1	Neg	1	3.66	4.42	4.58	5.18	23	4
Usidlenie	Ensnaring	1	Neg	1	3.88	4.90	4.52	5.38	9	9
Smarkacz	Stripling	1	Neg	1	3.36	4.88	4.64	3.46	265	8
Zaślepienie	Infatuation	1	Neg	1	3.32	3.66	4.40	5.64	75	11
Egzaminy	Exams	1	Neg	1	3.60	7.02	5.60	3.84	340	8
Ignorancja	Ignorance	1	Neg	1	2.98	6.14	4.68	6.44	140	10
Krata	Grating	1	Neg	1	3.98	6.16	3.60	1.74	357	5
Minus	Minus	1	Neg	1	3.84	6.36	3.52	4.90	554	5
Szpieg	Spy	1	Neg	1	3.92	6.72	4.20	3.30	637	6
Koszty	Costs	1	Neg	1	3.78	6.40	4.08	3.88	1134	6
Podwładny	Subordinate	1	Neg	1	4.12	6.28	4.02	4.06	189	9
Podatek	Tax	1	Neg	1	3.32	6.92	4.22	3.60	228	7
Alimenty	Alimony	1	Neg	1	3.60	6.34	4.48	3.42	82	8
Odsetki	Interest	1	Neg	1	3.78	6.56	4.36	3.80	58	7
Rząd	Government	1	Neg	1	3.80	6.50	4.50	3.64	5596	4
Przemyt	Smuggling	1	Neg	1	3.70	6.68	4.60	4.00	180	7
Recesja	Recession	1	Neg	1	3.63	6.65	4.20	5.13	25	7
Bezrobocie	Unemployment	1	Neg	1	2.92	6.06	4.20	5.40	122	10
Heretyk	Heretic	1	Neg	1	3.94	6.10	4.58	5.42	45	7
Procesja	Procession	2	Neu	2	4.76	4.88	3.50	3.56	293	8
Kościół	Church	2	Neu	2	5.24	4.46	3.54	3.78	3652	7
Kuksaniec	Nudge	2	Neu	2	4.53	4.55	4.10	3.02	13	9
Tarot	Tarot	2	Neu	2	4.16	4.90	3.72	3.92	38	5
Loteria	Lottery	2	Neu	2	5.76	4.70	4.10	3.46	56	7
Westchnienie	Sigh	2	Neu	2	5.48	4.28	3.60	4.44	1336	12
Jałmużna	Alms	2	Neu	2	4.36	4.84	4.04	3.92	44	8
Błazen	Clown	2	Neu	2	4.64	4.62	4.28	3.26	507	6
Mrowienie	Tingling	2	Neu	2	4.26	4.90	3.96	4.39	482	9
Pragnienie	Desire	2	Neu	2	5.14	3.40	5.18	5.80	4066	10
Obrzêd	Rite	2	Neu	2	5.04	4.76	3.66	4.90	220	6
Wróżka	Fairy	2	Neu	2	5.60	4.68	4.18	4.30	338	6
Młodzież	Youth	2	Neu	2	5.68	4.50	4.66	3.40	1703	8
Łasuch	Gourmand	2	Neu	2	5.74	4.72	4.40	4.00	9	6
Burza	Storm	2	Neu	2	4.86	4.54	5.30	3.06	3238	5
Szlachta	Nobility	2	Neu	2	5.46	6.22	4.08	3.90	811	8
Etykieta	Label	2	Neu	2	4.90	6.60	3.52	3.08	126	8
Sułtan	Sultan	2	Neu	2	5.10	6.84	3.46	3.10	397	6
Zadatki	Makings	2	Neu	2	5.32	5.98	3.80	5.34	94	7
Prawo	Right	2	Neu	2	5.84	7.60	3.68	5.96	19169	5
Prasa	Press	2	Neu	2	5.30	6.64	3.50	2.84	1232	5
Stawka	Bid	2	Neu	2	5.42	6.28	4.42	4.02	301	6
Raport	Report	2	Neu	2	4.88	6.98	3.56	2.84	2634	6
Wojsko	Army	2	Neu	2	4.94	6.62	4.96	2.90	2893	6
Interes	Business	2	Neu	2	5.86	7.10	4.36	4.82	3421	7
Dyscyplina	Discipline	2	Neu	2	5.46	6.44	3.96	5.74	384	10
Wynik	Result	2	Neu	2	5.52	6.70	4.60	4.58	1919	5
Weto	Veto	2	Neu	2	4.41	6.62	4.02	5.57	19	4
Hodowla	Breeding	2	Neu	2	5.56	6.12	3.62	2.82	131	7
Kurs	Course	2	Neu	2	5.48	6.66	3.48	3.82	2801	4
Zakochanie	Infatuation	3	Pos	3	7.56	2.28	6.50	7.24	52	10
Passa	Streak	3	Pos	3	6.16	4.84	4.36	5.78	41	5
Toast	Toast	3	Pos	3	6.36	4.60	4.30	3.92	689	5
Powitanie	Welcome	3	Pos	3	6.50	4.96	3.90	4.70	1825	9
Zapach	Fragrance	3	Pos	3	6.80	4.66	3.70	4.28	9963	6
Słodycz	Sweetness	3	Pos	3	7.02	4.44	4.14	3.54	477	7
Pomoc	Help	3	Pos	3	6.84	4.48	3.54	5.06	10180	5
Niemowlak	Infant	3	Pos	3	6.50	3.86	3.70	2.50	28	9
Flirt	Flirt	3	Pos	3	6.46	3.36	5.52	5.72	146	5
Potomstwo	Offspring	3	Pos	3	6.62	4.60	3.68	3.36	504	9
Pozdrowienie	Greeting	3	Pos	3	6.72	4.60	3.58	5.40	364	12
Skarb	Treasure	3	Pos	3	6.84	4.76	4.20	3.72	2460	5
Walentynka	Valentine	3	Pos	3	6.42	4.26	4.66	4.24	2	10
Podarunek	Gift	3	Pos	3	6.78	4.44	4.30	3.56	373	9
Ferie	Holiday	3	Pos	3	7.10	4.82	4.12	4.14	123	5
Miliard	Billion	3	Pos	3	7.08	7.06	4.68	4.18	311	7
Tolerancja	Tolerance	3	Pos	3	6.62	5.32	3.88	7.32	139	10
Mistrz	Master	3	Pos	3	7.22	6.24	4.52	4.38	4209	6
Patent	Patent	3	Pos	3	5.92	7.59	3.52	4.62	221	6
Dobytek	Property	3	Pos	3	6.48	6.76	3.74	3.78	552	7
Absolwent	Graduate	3	Pos	3	6.38	6.56	3.90	3.44	206	9
Uczony	Scholar	3	Pos	3	6.26	7.44	3.58	5.04	1421	6
Stypendium	Scholarship	3	Pos	3	7.06	6.62	4.12	3.16	372	10
Szczyt	Peak	3	Pos	3	6.44	6.12	4.08	3.28	3533	6
Równowaga	Balance	3	Pos	3	6.08	6.32	3.56	5.38	367	9
Oszczêdnośc	Savings	3	Pos	3	6.68	6.94	3.94	4.40	737	12
Płaca	Wages	3	Pos	3	6.16	6.82	4.27	3.27	63	5
Satyra	Satire	3	Pos	3	6.04	6.16	4.04	5.10	171	6
Lider	Leader	3	Pos	3	6.22	6.58	4.50	4.06	90	5
Zysk	Profit	3	Pos	3	6.78	6.88	4.18	4.76	651	4
